# Impact of connected health interventions on psychological wellbeing and quality of life in patients with cancer: A systematic review and meta‐analysis

**DOI:** 10.1002/pon.6019

**Published:** 2022-09-22

**Authors:** Isaiah Gitonga, Deirdre Desmond, Natalia Duda, Rebecca Maguire

**Affiliations:** ^1^ Department of Psychology Maynooth University Maynooth Ireland; ^2^ Assisting Living and Learning Institute Maynooth University Maynooth Ireland; ^3^ School of Psychology Trinity College Dublin Dublin Ireland

**Keywords:** cancer, connected health interventions, oncology, psycho‐oncology, psychological wellbeing, quality of life

## Abstract

**Objective:**

Connected health technologies have the potential to improve access to cancer care and support and reduce costs. We aimed to assess the impacts of interventions delivered using connected health technologies on psychological and quality of life (QoL) outcomes in people living with and beyond cancer.

**Methods:**

PUBMED, PsycINFO, Web of Science, and EMBASE were searched using terms relating to (i) cancer, (ii) connected health, and (iii) QoL/psychological wellbeing. Studies were included if they evaluated interventions using connected health technologies and assessed psychological and/or QoL outcomes for adults at any stage of cancer treatment or survivorship.

**Results:**

Thirty‐seven studies met the inclusion criteria with a total of 8956 participants. Connected health technologies included web‐based applications (*n* = 24), smart applications (*n* = 12), and wearable devices (*n* = 1). Studies were heterogeneous in terms of intervention components. We identified five clusters: (i) Psychosocial support and rehabilitation, (ii) psychoeducation and information support, (iii) symptom monitoring, reporting and self‐management, (iv) peer and social support, and (v) health coaching and physical activity training. Due to heterogeneity of outcome measures, the meta‐analysis included only seven RCTs; pooled mean estimates showed connected health interventions were moderately effective in reducing symptoms of depression (SMD: −0.226, 95% CI −0.303/−0.149) and anxiety (SMD: −0.188, 95% CI: 0.279/−0.0963) compared with usual care.

**Conclusion:**

While the considerable heterogeneity observed highlights the need for more rigorous studies to improve reproducibility and efficiency, results suggest that connected health interventions have the potential to improve psychological wellbeing and QoL outcomes in people living with and beyond cancer.

## 1 BACKGROUND

Advances in cancer screening, diagnosis and treatment have resulted in an increasing number of people living with and beyond cancer (LWBC).^1^, ^2^ Given the range of symptoms that can be experienced,^3^ there is a clear need to identify ways to enhance quality of life (QoL) in this group.^2^, ^4^ Beyond the effects that cancer can have on physical health, it may have an even greater and, arguably, more significant impact, on mental health.^5^, ^6^ Feelings of uncertainty, fear, or sadness resulting from diagnosis are associated with increased psychological distress,^7^ which may interfere with coping strategies.^8^ While effective management of symptoms can reduce distress, enhance coping, and improve QoL,^9^ psychological wellbeing remains a top unmet need for people LWBC.^10^

Psychosocial interventions such as cognitive behavioural therapy (CBT) and behaviour change techniques can enhance coping skills and improve QoL in people LWBC.^11^, ^12^ However, person‐to‐person interventions can be costly and hard to access, especially for individuals in hard‐to‐reach areas, those working or with caring responsibilities.^13^ Rising cancer incidence and a shrinking healthcare workforce may exacerbate challenges,^13^, ^14^ with COVID‐19 most recently presenting obstacles for in‐person care.^15^

Technology‐based approaches to care, such as connected health (CH), may help overcome challenges by facilitating increased access to individualised support.^4^, ^16^ CH is a fast‐growing paradigm in healthcare innovation where devices, interventions and services are designed around patients' needs through efficient data collection, analysis, and transfer.^17^, ^18^ CH is considered an umbrella term to reduce confusion over definitions of telehealth, telemedicine and mobile health.^17^ CH entails technologies that are predictive, pre‐emptive, personalised, patient centric and participatory.^18^ It differs from other technologies in that a two‐way flow of information is used.^19^

The impact of CH in cancer care outcomes has been evaluated in previous reviews with mixed, albeit promising, evidence. For example, CH was found effective in enhancing patient engagement,^20^ self‐management,^21^ and reducing barriers to care.^22^ Other reviews evaluated design features of CH in cancer care,^23^ its utility in cancer follow‐ups,^24^ effects on wellbeing and QoL outcomes,^25^ and the benefits in supportive care.^26^ However, these reviews reported mixed findings, acknowledging a lack of quality evidence regarding the efficacy of CH technologies. A recent review noted the need for more high‐quality trials, especially those using standardized outcome measures.^27^

CH technologies are acceptable among cancer patients, particularly those including elements of social support, self‐management, and remote access to professionals.^28^ However, while CH can enhance outcomes such as self‐efficacy, coping, and perceived social support,^27^, ^28^, ^29^ there is limited evidence for its impact on severe symptoms of psychological distress, such as anxiety or depression.^25^ Additionally, the impact of CH on other psychological and QoL outcomes in people LWBC remains unclear.

Previous reviews have restricted their evaluations to specific subsets of CH,^27^, ^28^, ^29^ noting a shortage of interventions and study heterogeneity. Thus, a quantitative analysis of CH efficacy is needed. Given the sharp increase in CH interventions over the last decade, rapid shifts in technologies, and the increased demand for remote services in the context of the COVID19 pandemic, it is critical that their efficacy is continuously evaluated.^30^ The present review aimed to assess the impact of interventions delivered using CH technologies on psychological and QoL outcomes in cancer.

## 2 METHODS

This review was conducted in compliance with the Preferred Reporting Items for systematic review and meta‐analysis (PRISMA) guidelines. The protocol is registered with the Prospective Register for Systemic Reviews Database (ID: CRD42021246828).

### 2.1 Search strategy

Searches were completed in May 2021 to identify articles pertaining to CH interventions for people LWBC. Any study evaluating a CH‐facilitated intervention and reporting psychological wellbeing and/or QoL, either as a primary or secondary outcome, published in a peer‐reviewed journal and in the English language was deemed eligible for inclusion (see Supplementary material S1). Only technologies that were ‘connected’ and offered a two‐way communication in the flow and use of data were included.^17^, ^18^, ^19^ Considering the technological advancements in the last decade, only studies published in the past 10 years were considered. Bibliographic mining and citation searching of studies obtained were also conducted.

Studies were identified by searching electronic databases (PubMed, PsychINFO, Web of Science, EMBASE) using terms relating to (1) Cancer, (2) QoL/Psychosocial Wellbeing and (3) CH. (See Supplementary Material S1 for full syntaxis). Search terms were developed by IG, RM and DD based on previous literature.^31^, ^32^ Boolean operators were employed to search the selected databases. MeSH, EMTREE, PsycINFO thesaurus or equivalent terms were used and exploded.

### 2.2 Screening

Results of database searches were exported to Endnote and duplicates removed. A standardized online platform Rayyan^33^ was used to screen studies. Title and abstract screening was completed by two reviewers (IG and ND) independently. The remaining articles underwent full‐text reviews by independent reviewers to confirm eligibility. Disagreements were discussed amongs authors until consensus was obtained. Available data for aggregation were required for inclusion in the meta‐analysis.

### 2.3 Data extraction

The following data were systematically extracted by IG (checked by ND) and inputted into an Excel spreadsheet: author, year, title, design, number/characteristics of participants, including cancer type, intervention type, outcome measures, results obtained, and study limitations. If required data were not reported, the corresponding author was contacted to obtain this or to seek additional details.

### 2.4 Methodological quality assessment

IG and ND independently conducted a quality assessment for included studies using the Mixed Methods Appraisal Tool (MMAT).^34^ The MMAT is intended to critically assess the quality of quantitative, qualitative, randomized controlled trials (RCT), non‐randomized and mixed methods studies. This consists of two screening questions followed by five design‐specific questions. Conflicts in quality assessments were resolved through discussion until consensus was reached. The latest MMAT guidelines discourages presenting a single number to denote quality as it does not tell what specific study areas are problematic.^34^ For this reason, interpretation took the following form: 4–5 criteria met = high quality, 2–3 criteria met = moderate quality, 0–1 criterion met = low quality, as per previous analysis.^34^

### 2.5 Synthesis of findings

Study characteristics, interventions, and outcomes were described in table form. A preliminary analysis was employed to assess the nature of data available for meta‐analysis. Considering the heterogeneity in outcomes variables and measures, thematic synthesis was deemed suitable to summarise the evidence.^35^ This enabled us to aggregate evidence regarding the impact of CH on psychological wellbeing/QoL and to identify patterns within data relating to these outcomes. We synthesized findings in three stages. First, data pertaining to psychological wellbeing/QoL outcomes from CH‐facilitated interventions were coded. Here, the primary reviewer developed a coding frame derived from the data, which was reviewed by the other reviewers, with discrepancies resolved through discussion. Next, similarities between codes were identified. Codes were grouped into themes that captured outcomes/patterns across included studies. Each theme was entered as a separate column in a table, while coded data from each study were entered in rows to illustrate themes. This technique facilitated constant comparison within and between studies as part of the constant comparison analytic process. Finally, analytic themes were developed with the aim of synthesizing findings across studies and interpreting their meaning in terms of psychological wellbeing and QoL. This included a narrative description of each theme, enabling results to be aggregated, thus providing an overall account of CH impact on reported psychological/QoL outcomes in people LWBC. The focus of interventions was summarized into five thematic areas: (i) Psychosocial support and rehabilitation, (ii) psychoeducation and information support, (iii) symptom monitoring and self‐management, (iv) peer and social support, and (v) health coaching and physical activity training. Studies within each of these clusters were evaluated based on reported psychological and QoL outcomes.

### 2.6 Measures of intervention effect

Only seven studies had complete data for inclusion in the meta‐analysis. Outcome measures of included studies were all continuous and reported on the Hospital Anxiety and Depression (HADS) scale,^36^ therefore standardized mean difference (SMD) and standard error (SE) were used to summarize estimates of effects from individual studies.^37^ The magnitude of standardized mean differences was interpreted using Cohen's conventions for small (SMD = 0.2), medium (SMD = 0.5), and large (SMD = 0.8) effects.^38^ Since considerable heterogeneity was expected, we chose a random‐effects pooling model for all analyses a priori.

### 2.7 Assessment of heterogeneity

Inconsistency between study estimates was both visually and statistically examined through inspection of forest plots and consideration of the I^2^, respectively. The I^2^ was calculated to assess heterogeneity. In general, heterogeneity was categorized as low (0%–40%), moderate (30%–60%), substantial (50%–90%), or considerable (75%–100%).^39^ To examine small study effects, funnel plots and the Egger's test was used. Data available for meta‐analysis was analysed using R software.

## 3 RESULTS

**FIGURE 1:**
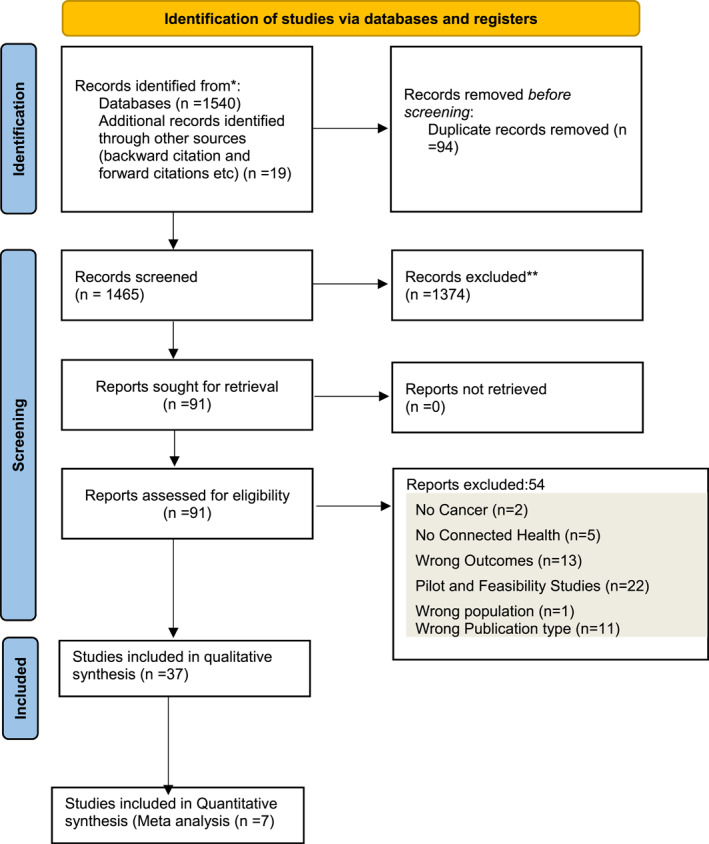
Preferred Reporting Items for Systematic Reviews and Meta‐Analysis (PRISMA) diagram

Database searches yielded 1446 articles for title and abstract screening following duplicate removal. After initial screening, 90 full texts were assessed for eligibility, with 54 excluded (Figure 1). An additional 19 papers were identified through other sources such as manual forward and backward citation chasing and trial registry search leading to 37 studies included. Of these, 36 were controlled trials, with 35 being randomized and one nonrandomized. One study utilized a qualitative methodology. Of the 36 trials, nine^40^, ^41^, ^42^, ^43^, ^44^, ^45^, ^46^, ^47^ used HADS as a measure anxiety and depression symptoms and were considered for meta‐analysis. However, two studies^40^, ^41^ were not included because complete data were not available. EORTC Core QoL questionnaire (EORTC QLQ‐C30)^31^ was most frequently used to assess different aspects of QoL depending on the cancer type, with other studies measuring different psychological wellbeing/QoL outcomes. Due to heterogenous outcomes and measures for QoL, a meta‐analysis was not appropriate. All studies were included in the qualitative synthesis.

Studies included 8956 survivors in total (mean age = 44–69 years). Sixteen studies evaluated impact of interventions in the survivorship phase, while the rest were evaluated either in active treatment or across active treatment and survivorship phases. Participants in included studies had various types of cancer namely breast (*n* = 15), haematological (*n* = 2), colon (*n* = 1), nasopharyngeal (*n* = 1), prostate (*n* = 1), lung (1), and mixed/multiple cancers (*n* = 16) (Supplementary 1).

### 3.1 Quality appraisal

Variability in methodology and study quality were noted (Table 1). Fourteen studies met 4–5 criteria (high quality) while the remaining 23 met 2–3 criteria (moderate quality). Frequent limitations related to the randomization processes, non‐blinded outcome assessors, non‐representative samples, and non‐adherence to interventions.

**TABLE 1:** Intervention outcomes

Study	Design	Intervention description	Control	Intervention duration	CH intervention components	Outcomes	MMAT
Owen et al.^74^	RCT	Social networking Platform *[The Health‐Space Intervention (* health‐space.net *)]*	Waitlist control (WC)	12 weeks	Self‐management, peer support, psychosocial support, coping skills training	Lower clinically significant depression in both conditions. Intervention did not improve depression, trauma‐related anxiety symptoms, or overall mood disturbance	3
Yun et al.^42^	RCT (multi‐centred)	Web‐based support with health coaching and Web‐based support without health coaching	Health education booklet	6 months	Self‐management, information support, health coaching	Greater reduction in anxiety in intervention than control. No differences in depression across the three groups	3
Beatty et al.^67^	RCT	Self‐guided web‐based CBT *(Cancer Coping Online)*	Web‐based information only	6 weeks	Psychoeducation, psychosocial support (CBT‐based activities)	Lower cancer distress at 6‐month follow‐up and higher global QOL in intervention than control	4
Abrahams et al.^66^	RCT	Internet‐based CBT (ICBT)	Care as usual (CAU)	6 months	Psychosocial support (CBT activities)	Lower functional impairment and psychological distress, and higher QOL for intervention	5
Lally et al.^55^	RCT	Unguided, web‐based, psychoeducational program (CaringGuidance™ After Breast Cancer Diagnosis)	CAU	12 weeks	Self‐management, psychoeducation, information support	No overall effects but lower depressive symptoms and distress differences between months 2 and 3 in intervention	4
Greer et al.^44^	RCT	Mobile‐based (app) CBT	mhealth education program	12 weeks	Psychosocial support (CBT activities), psychoeducation	Improvements in anxiety, depression, and QOL for both groups. No differences in outcome measures but secondary analysis showed mobile CBT group had less anxiety compared with control	3
Stevenson et al.^47^	RCT	Web‐based information tool and nurse‐delivered telephone support	CAU	12 weeks	Information support	No differences in unmet information needs, depression, or anxiety between groups. Decrease in unmet information needs in both groups	4
Sherman et al.^69^	RCT	Web‐based psychological intervention (structured online writing exercise) plus usual care	Expressive writing plus CAU	1 week	Psychosocial support	Lower body image distress and psychological distress in intervention	5
Spahrkäs et al.^61^	RCT	Self‐management mHealth app *(Untire App).*	WC	12 weeks	Psychosocial activities (CBT), Psychoeducation, Physical activity training	Greater improvements in average overall QoL but not for overall QoL in the past week for intervention	3
Sui et al.^46^	RCT	WeChat app‐based education and rehabilitation program	Simple education and rehabilitation guidance	12 months	Health education, Rehabilitation, activity supervision, psychosocial support	Lower anxiety scores, anxiety rate, depression scores, and depression rate in intervention. Higher QLQ‐C30 global health status score and functional score, but no difference in QLQ‐C30 symptom score in the intervention compared with control	5
Beatty et al.^67^	RCT	Online self‐guided psychotherapeutic intervention *(Finding My Way)*	Information‐only	6 weeks	Psychosocial support, psychoeducation	Both groups reported reduced cancer‐specific and general distress over time, with no group differences	4
Ruland et al.^58^	RCT	WebChoice: Internet‐based, interactive health communication application	Information sheet with suggestions for publicly available, cancer‐relevant Internet sites	12 months	Self‐assessment, self‐management, information support, peer/social support	No group differences in depression and HRQoL, with only global symptom distress index on MSAS‐SF lower in intervention	4
Zhou et al.^63^	RCT	WeChat‐based multimodal nursing program plus routine nursing care	CAU	6 months	Peer/social support, psychosocial rehabilitation	Improvement in HRQOL in intervention group	5
Korkmaz et al.^56^	RCT	Web‐based Patient education Or Brochure group	CAU	40 days	Health education	The differences in the state of anxiety scores were statistically lower in the web‐based education group than in the brochure and CAU group	3
Bruggerman‐Everts et al.^41^	RCT	Psychologist‐guided web‐based mindfulness‐based CBT (Embct) or Psychotherapist guided Ambulant Activity Feedback	Psychoeducational Emails	9 weeks	Psychosocial support, psychoeducation, Physical activity support	Fatigue severity decreased more in intervention groups compared to control. Mental health improved in all groups	4
Willems et al.^43^	RCT	Stand‐alone web‐based psychosocial intervention [Kanker Nazorg Wijzer (KNW; Cancer Aftercare Guide)].	WC	12 months	Psychosocial, self‐management, information support	Intervention effective in improving social functioning for men, reducing fatigue for participants ≤56 years, and depression for participants who received chemotherapy at 6 months. Effects not sustained at 12 months	3
Rosen et al.^64^	RCT	Mobile app‐delivered mindfulness training (AMT) (Headspace)	WC	8 weeks	Psychosocial Support (mindfulness training)	Higher QOL in intervention than control from baseline through follow‐up	3
Kuhar et al.^71^	RCT	Mobile App (mPRO Mamma)	CAU	Varied	Symptom monitoring/management	Summary global QoL higher for intervention than control after first week and at end of treatment	5
Vallance et al.^75^	RCT	ACTIVity And TEchnology (ACTIVATE*)‐ wearable technology‐based intervention*	WC	12 weeks	Physical activity rehabilitation, psychosocial/behaviour change facilitation	No HRQoL differences between groups but small improvement in fatigue at T2	3
Anja van der Hout et al.^49^	RCT	Web‐based eHealth application (Oncokompas)	WC	6 months	Behaviour change, information support, self‐efficacy, self‐management confidence	Improvements in HRQoL and tumour‐specific symptom burden. No differences in patient activation between groups over time	3
Fjell et al.^60^	RCT	Interactive smartphone application (Interaktor)	Standard care (SC)	Varied	Self‐assessment, symptom monitoring, information support	Lower overall symptom distress and physical symptom distress, and higher emotional functioning in intervention group	4
Sundberg et al.^65^	CT (non‐Randomized)	Interactive smartphone application *(Interaktor)*	CAU	5–8 weeks	Self‐assessment, symptom monitoring/management	Lower levels of fatigue, nausea, burden on emotional functioning, insomnia, and urinary‐related symptoms in intervention group at end of radiotherapy and 3 months later	2
Compen et al.^40^	RCT	Face‐to‐Face MBCT Or eMBCT	CAU	3 months	Psychosocial support (mindfulness‐based CBT), information support	Less psychological distress in intervention groups, with reduced fear of cancer recurrence, increased mental HRQoL mindfulness skills, and positive mental health compared with TAU. No improvements in physical HRQoL	3
Syrjala et al.^54^	RCT	Internet‐based Survivorship Program with Information and REsources, with Problem‐Solving Treatment (PST) telehealth calls (*INSPIRE)*	WC	6 months	Psychosocial support (Problem‐solving therapy), Information support	No reduction in aggregated outcomes for either intervention. INSPIRE + PST participants were more likely to improve in distress than controls, with INSPIRE alone marginally more likely to improve in distress	3
Ridner et al.^52^	RCT	Web‐based Multimedia Intervention (WBMI)	Educational pamphlets	12 modules, each lasting 30 mins	Psychosocial support, information support	Group differences in symptom reduction between baseline and 1/12 months, apart from mood symptoms	3
van Helmondt et al.^48^	RCT	CAncer REcurrence Self‐help Training (CAREST): CBT‐ based online tailored self‐help training	CAU		Psychosocial support, psychoeducation	No differences between groups suggesting treatments did not differ in their change in fear of cancer recurrence over time	2
Chambers et al.^53^	RCT	CBT‐based online self‐help training (*CancerCope program)*	CAU	6 weeks	Psychosocial support, self‐assessment, management	No significant intervention effects on fear of cancer recurrence, psychological distress, or other outcomes. Analysis showed a greater decrease in psychological distress, cancer‐specific distress and unmet psychological care needs from baseline to 8 weeks in intervention group compared with the patient education group	2
Hou et al.^74^ (2020)	RCT	Breast cancer self‐management support mHealth (BCSMS) app.	CAU	6 months	Self‐management, information support	Mean total QoL summary scores from the QLQ‐C30 significantly higher among experimental group versus the control group at 3 months	3
Hauffman et al.^51^	Qualitative	*‘iCAN‐DO’:* internet‐based stepped‐care program	NA	10 weeks	Psychosocial and information support	The intervention was experienced as a useful and reliable source of information and support and was used as a complement to standard care	5
Li and Di^72^	RCT	Smartphone medical app after discharge.	CAU	Varied	Self‐management, information support, link to experts	QoL was higher in intervention group than in the control group at 6 months	2
Admiraal et al.^57^	RCT	Web‐based tailored *psychoeducational program (ENCOURAGE)*	SC	12 weeks	Self‐assessment, self‐management, psychoeducation	Study groups did not differ across outcome measures	3
Willems et al.^50^	RCT	Web‐based psychosocial intervention *[Kanker Nazorg Wijzer (KNW; Cancer Aftercare Guide)]*	WC	6 months	Self‐management, information support	Intervention effective in reducing depression and fatigue levels	4
Mayer et al.^62^	RCT	SurvivorCHESS (Comprehensive Health Enhancement Support System): a smartphone application	Educational booklets	6 months	Skills building, psychosocial support, physical activity	No differences between groups over time for QOL or distress items. At 6 months, physical activity in intervention group improved from moderate to vigorous but improvement not sustained 3 months after study ended	3
Greer et al.^59^	RCT	Smartphone app and Fitbit integration for tracking physical activity	SC	12 weeks	Symptom monitoring/management, physical activity tracking	Study groups did not differ across outcome measures	3
Freeman et al.^69^	RCT	Envision the Rhythms of Life (ERL): imagery‐based group intervention either Live‐delivery versus therapist streamed via telemedicine	WC	3 months	Psychosocial support, social support, self‐assessment	Clinically significant improvements in fatigue, cognitive dysfunction, sleep disturbance, health‐related and breast cancer‐related QOL in intervention groups compared to control after 3 months. No differences between live and telemedicine‐delivered interventions	3
Yun et al.^45^	RCT	Internet‐based, individually tailored CRF education program	CAU	12 weeks	Psychosocial support, information support, physical activity rehabilitation	Decrease in anxiety scores, global QoL, and several functioning scores of EORTC QLQ‐C30 in intervention group	3
Basch et al.^73^	RCT	Web‐based Symptom Tracking and Reporting (STAR)	CAU	6 months	Symptom tracking and reporting	HRQoL improved among more participants in the intervention group than control group	3

### 3.2 Intervention characteristics

The duration of interventions and measurement points varied. Twelve studies had one follow‐up, while the rest had multiple follow‐ups (range: 2–5). Ten studies had a waitlist control, 11 had active controls, and 15 used care as usual. Of 36 controlled studies, 24 evaluated interventions that included contact with health care providers or physical activity coaches. These included nurses (*n* = 8), trained coaches (*n* = 2), oncologists (*n* = 4), trained therapists and psychologists (*n* = 10). Others were unguided or self‐guided. (Supplementary material).

### 3.3 Connected health technologies

In terms of CH technologies, 21 interventions utilised web programs, including web‐based self‐guided psychosocial interventions (*n* = 8), web‐delivered CBT and mindfulness sessions (*n* = 8), and web‐based psychoeducational programs (*n* = 5). Thirteen interventions used smart applications with symptom monitoring, self‐assessment, and self‐management programs (*n* = 9), and those that facilitated social networking (*n* = 4) such as WeChat. Two studies evaluated live therapist streamed sessions via videoconferencing software, while one evaluated a wearable device. Only one‐sixth (*n* = 6) of included CH technologies were publicly available platforms/websites.

#### 3.3.1 Intervention outcomes

Impacts of interventions were categorized based on outcomes in psychological wellbeing and QoL domains. For psychological wellbeing, patient‐reported outcomes included depression, anxiety, and symptom distress, while QoL outcomes included HRQoL, physical activity, and fatigue. Several studies evaluated multiple outcomes. Impacts of interventions on various outcomes are discussed in terms of the thematic clusters of interventions identified, with a select number of studies described as illustrative examples (Table 1).

##### Psychoeducation and information support

Twenty‐six studies evaluated the impact of CH‐delivered psychoeducation and information support on distress and anxiety. Technologies included web‐based psychoeducation and information support programs^40^, ^41^, ^42^, ^43^, ^45^, ^47^, ^48^, ^49^, ^50^, ^51^, ^52^, ^53^, ^54^, ^55^, ^56^, ^57^ or interactive health communication applications.^44^, ^46^, ^58^, ^59^, ^60^, ^61^, ^62^, ^63^, ^64^, ^65^ These included dedicated information where users could access reliable and relevant web resources related to their illness, allowing them to stay connected with health care providers to address concerns that would cause undue anxiety and distress. There were mixed efficacies. One web‐based information tool and nurse‐delivered telephone support was intended to reduce unmet information needs, depression, and anxiety among haematological cancer patients, however this did not yield any differences in comparison to usual care.^47^ Similarly, when compared to usual care, a web‐based, psychoeducational distress self‐management program, CaringGuidance™ After Breast Cancer Diagnosis, found no significant overall effects post‐intervention.^55^ Another web‐based patient education intervention for patients hospitalized following breast surgery showed state anxiety was lower at three time points compared to control groups,^56^ while a qualitative study exploring user experiences of internet‐based stepped care (iCAN‐DO) in patients with concurrent symptoms of anxiety and depression reported that finding information was considered a “survival strategy” to reduce symptoms of anxiety and depression when receiving a cancer diagnosis, suggesting that this intervention was helpful in reducing symptoms.^51^

##### Psychosocial support and rehabilitation

Half of interventions (*n* = 18) targeted psychosocial support and rehabilitation, measuring the impact on various domains of psychological wellbeing and QoL. Interventions encompassed web‐based CBT,^40^, ^41^, ^43^, ^48^, ^50^, ^53^, ^66^, ^67^, ^68^ mobile‐based CBT,^44^, ^47^, ^61^ WeChat app‐based education and rehabilitation program (WERP),^46^, ^57^, ^63^ Web‐based psychologist‐guided interventions,^69^ mobile app‐delivered mindfulness training (AMT),^64^ and web‐based Multimedia Interventions (WBMI).^45^, ^52^, ^70^ Topics covered included self‐care, goal setting, self‐reward, dealing with negative feelings and building social support. Mixed efficacies were reported.

One study examined the efficacy of a tailored CBT mobile application compared with mobile health education to treat anxiety in patients with incurable cancer.^44^ While groups did not differ in improvements in anxiety, depression, and QoL, the CBT intervention was more beneficial for patients with severe baseline anxiety. In a related study, breast cancer survivors in the ‘My Changed Body (MyCB)’, a Web‐based psychological intervention, reported less body image distress and greater body appreciation and self‐compassion than expressive writing survivors (control).^70^ An internet‐based Mindfulness‐CBT intervention led to less psychological distress, reduced fear of cancer recurrence and improved HRQoL, mindfulness skills, and positive mental health compared with treatment as usual, but no improvements in physical QoL.^40^ An Imagery‐based Behavioural Intervention for Breast Cancer Survivors “Envision the Rhythms of Life” (ERL)’ resulted in clinically significant improvements in multiple QoL domains compared to a waitlist control.^69^

##### Symptom monitoring, reporting, and self‐management

Eighteen studies examined the role of CH interventions on symptom burden, symptom monitoring, and self‐management and evaluated the resultant impact on psychological wellbeing and QoL outcomes. Most studies (*n* = 13) examining symptom management were based on smart applications,^44^, ^46^, ^59^, ^60^, ^61^, ^62^, ^63^, ^64^, ^65^, ^71^, ^72^, ^73^, ^74^ while one employed a wearable device.^75^ These interventions involved a symptom assessment section for patients, and tailored symptom self‐management support. Mixed findings on psychological wellbeing and QoL were reported. One mobile app ‘MPRO mamma’ to support symptom management and associated QoL in early‐stage breast cancer^72^ involved daily tracking of symptoms, allowed users to grade symptom severity, and provided in‐depth descriptions and recommendations based on reported symptom levels. This was associated with better QoL compared to the control. Separately, an interactive smartphone application ‘Interaktor’ was associated with lower levels of fatigue and nausea at the end of radiotherapy, and less burden in emotional functioning, insomnia, and urinary‐related symptoms among prostate cancer patients at the end of treatment and 3 months later.^65^ A web‐based symptom tracking and reporting (STAR) intervention resulted in improvements in HRQoL in patients receiving routine outpatient chemotherapy compared to care as usual.^75^ Another study found differences in global symptom distress index between the ‘web choice’ intervention and control groups, but no differences in depression, self‐efficacy, and HRQoL.^58^

##### Peer and social support

One quarter (*n* = 9) of included studies involved CH‐mediated peer support and social networking interventions. Patients could share experiences with other patients and obtain professional support. In addition, users had access to a support forum for group discussion allowing them to ask questions and share experiences in the comfort of their homes and with confidentiality. The impact of these interventions on psychological and QoL outcomes was evaluated with overall promising efficacy. One study evaluated the effects of a 12‐week social networking intervention ‘healthspace.net’ on distress, depression, anxiety, vigour, and fatigue in cancer survivors reporting high levels of cancer‐related distress. Post‐intervention, the prevalence of clinically significant depression symptoms declined from 67% to 34% in both groups.^76^ A WeChat‐based multimodal led to significant improvements in HRQoL among postoperative breast cancer patients.^63^ Additionally, a 12‐month WeChat‐based education program was found effective in improving wellbeing and QoL in non‐small lung cancer patients after undergoing surgical resection.^46^

##### Health coaching and physical activity training

Three CH interventions targeted health coaching, skills training and physical activity with a working hypothesis that improved physical activity after diagnosis may decrease recurrences and improve QoL and physical functioning.^41^, ^62^ Survivors of colon cancer using ‘SurvivorCHESS’, increased their moderate to vigorous physical activity, but this was not sustained 3 months post‐intervention, with no QoL or distress differences over time.^62^ The ACTIVATE Trial examined the efficacy of a wearable‐based intervention to increase physical activity in breast cancer survivors.^75^ A 4.6‐point difference in fatigue was observed between groups at the end of intervention indicating improvement in fatigue profiles in the intervention group, with no effects on HRQoL. In a related study, a self‐management mHealth app “Untire mobile app’’ improved fatigue and QoL, with larger improvements in fatigue severity fatigue interference and overall QoL.^61^

#### 3.3.2 Meta‐analysis findings

**FIGURE 2:**
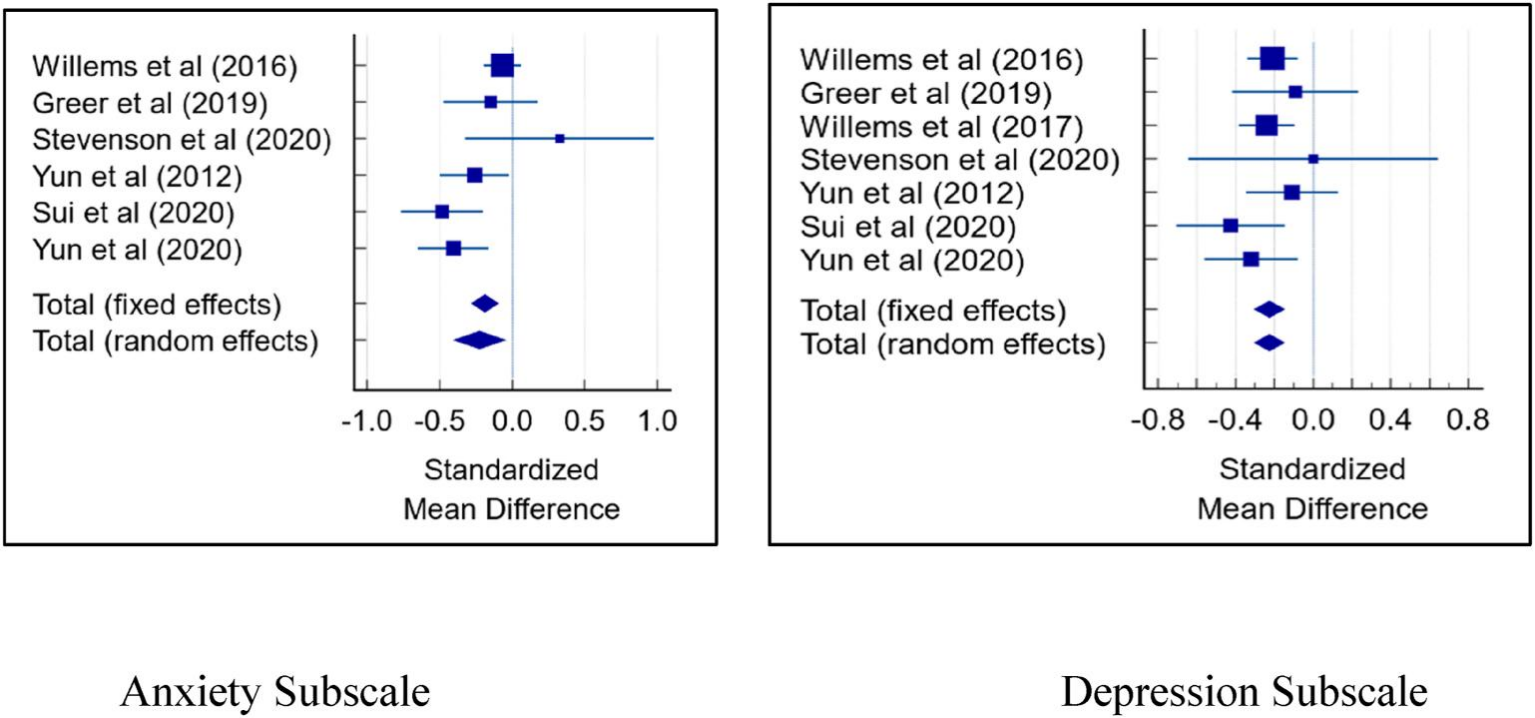
Effects of interventions on anxiety and depression (HADS)

Seven trials^42^, ^43^, ^44^, ^45^, ^46^, ^47^, ^50^ evaluating the impact of CH interventions on psychological outcomes of anxiety and depression using HADS were included in the meta‐analysis. One trial^43^ reported HADS‐ depression subscale only so was not included. Pooled estimates of both depression and anxiety scores from HADS showed that CH interventions were moderately effective for depression (SMD: −0.226, 95% CI −0.303/−0.149) and anxiety (SMD: −0.188, 95% CI: 0.279/−0.0963) compared to controls. The overall *I* ^2^ (inconsistency) was 63.7% for anxiety, indicating moderate heterogeneity. No heterogeneity was observed for depression. We did not find significant publication bias based on the insignificant Egger's test and through funnel plot examination. Figure 2 shows the effect on interventions on the HADS subscales of anxiety and depression.

## 4 DISCUSSION

This review examined CH‐mediated interventions on psychological wellbeing and QoL outcomes in cancer. Depression and anxiety were the most‐commonly evaluated psychological outcomes, with findings suggesting potential for CH to improve wellbeing of those LWBC. This is consistent with previous reviews that found depression and anxiety as top psychological concerns and targets for CH interventions in cancer.^29^, ^77^ Overall, we also found a generally positive effect of interventions on QoL. However, while evidence suggests benefits for CH in all five clusters reviewed, the diversity of interventions and outcome measures call for more evidence‐based evaluations.

The studies reviewed used a wide range of CH technologies, with content similar to that of traditional face‐to‐face interventions. Results indicate that psychosocial and behaviour change interventions perform well when delivered via CH, with equal or higher efficacy compared to usual care. This could help in reducing travel burden, commonly reported as barrier to cancer care.^13^ Our review evaluated a diverse spectrum of CH interventions, with many trials evaluating multiple outcomes in different cancer phases. While this is encouraging, it may not be possible to associate specific outcomes with specific intervention components, thus possibly diluting effects. This concern has been consistently noted in previous reviews.^23^, ^78^ suggesting future studies should aim to assess specific CH components and outcomes separately to maximize efficiency.

A considerable number of trials investigating CH interventions show the promising role of CH in supporting psychological wellbeing. To our knowledge, this is the first study to attempt a meta‐analysis, albeit with a limited selection of outcomes. However, like previous reviews,^23^, ^78^, ^79^ thematic synthesis revealed varying efficacies on different measures of psychological wellbeing. Mixed findings reflect the diversity of outcome measures and heterogeneity of studies in terms of sample compositions and mechanisms through which improvements were proposed to occur.^4^, ^80^ Mixed findings may also be because the ‘usual care’ in studies differed considerably (ranging from active controls, waitlist control or person‐to‐person care). Thus, additional research is needed to understand the optimal timing and delivery of interventions through standardised control conditions.

It is important to note that most records identified from the initial database searches were pilot and feasibility studies, which were excluded from this review given our focus on full‐scale interventions. The increased demand for remote services and accelerating pace of CH technologies in response to the COVID‐19 pandemic^81^ is a likely driver of the increase in such trials, further indicating the need for continued evaluation of efficacy.

Our review found promising efficacy of emerging technologies such as wearable devices and social networking platforms in supporting psychological wellbeing and QoL. The 12‐week social networking application by Owen et al.^76^ reported improvements in distress that were not associated with severe anxiety symptoms, while the 12‐month WeChat app‐based program by Sui et al.^46^ reported higher reductions in anxiety and depression compared to control, with overall improvements in QoL in both groups. Vallance et al's^75^ wearable technology reported small improvements in fatigue, but no effects on overall HRQoL. Taken together, the findings suggest that emerging technologies may be useful in improving certain outcomes for psychological wellbeing and QoL but may not be effective among patients with severe mental disorders.

Studies included in our review evaluated the impact of CH based interventions across different phases of cancer, with more being conducted among survivors rather than those in active treatment. While CH intervention targets were largely similar across cancer care phases, CH was found useful in symptom monitoring to reduce symptom related distress in the active treatment phase, while cancer related distress, body image distress, QOL and fear of recurrence were largely targeted in the post treatment phase. These concerns have been reported as among the unmet needs in various phases.^1^, ^2^, ^10^

Considering improvements in therapeutic interventions for cancer in the last decade,^1^, ^2^ this finding is not surprising, with more people LWBC. Notably, the meaning of ‘cancer survivor’ varied across contexts. In some contexts, survivorship was defined as the post‐treatment period of care with a focus on ‘cured’ or having completed active treatment with curative intent but excluding end‐of‐life care, while other studies defined a cancer survivor as an individual from the time of diagnosis through the end of life. There is need for consensus on the meaning of survivorship across all contexts.

Even though the majority of studies reported considerable adherence to interventions with an intention to treat analysis, a finding consistent with other studies on CH technologies,^82^ further research is needed to understand the main components and delivery approaches to maximise patient engagement. The large variability in intervention durations was another notable finding, with some lasting as short as a few days to others taking several months. This highlights the need to identify the most effective durations for CH interventions. Only one study explored user experiences of internet‐based stepped care in patients with cancer and concurrent symptoms of anxiety and depression.^51^ Very few explored patients' reasons for using or not using CH interventions. There is a need for further studies targeting non‐users to improve uptake of CH, which holds promise in the context of increasing challenges in face‐to‐face interventions.

On a positive note, the quality of the included studies was generally high. This is an additional strength indicating an increasing standard of evidence for CH interventions and an improvement from a previous systematic review^27^ noting a general lack of high‐quality primary studies and RCTs. However, the lack of standardised outcome measures remains a major concern.

### 4.1 Study limitations

There are several limitations to the present review. Firstly, considering CH is a developing concept in digital health with a somewhat broad definition, the lack of consistent terminology may have hampered article identification for analysis. Secondly, pilot and feasibility studies were excluded and considering many of them might have deployed to offer remote services during COVID19 pandemic, it is possible that additional technologies with improved efficacy have been more recently developed. On the flip side, this reflects the urgent need for further examination of CH in line with the recent call from WHO for enhanced evaluation to inform integration and use of digital technologies.^30^ Thirdly, the included studies may not comprehensively represent CH technologies and cancer subtypes as they were incidental to psychological wellbeing and QoL outcomes. As such, any conclusions should be tentative in light of the likely partial data. Finally, only reports in English were included, thus excluding studies published in other languages.

### 4.2 Clinical and policy implications

Our results suggest CH can be incorporated into clinical practice to manage psychological concerns in people LWBC. CH could be clinically useful for patients experiencing mild to moderate symptoms of depression and anxiety. At a policy level, more research and investments are required from all stakeholders, including user involvement in design to improve uptake, mass rollout, and sustainability, particularly in the aftermath of the COVID‐19 pandemic. However, potential concerns of CH use may exist, such as data privacy and security, with unintended consequences such as widening inequalities attributable to the existing digital divide as a result of low education, income, and poor connectivity,^4^, ^16^ especially in low resources and underserved settings where digital literacy and supporting infrastructure remains a barrier.^4^, ^16^, ^30^

## 5 CONCLUSION

CH‐mediated interventions have the potential to support psychological wellbeing and improve QoL in cancer survivorship. However, future rigorous research employing both qualitative and experimental designs is needed to comprehensively inform the relevant components, timing, design, intensity and delivery of these interventions, particularly for the emerging technologies. Additionally, research examining the generalisability of CH should be conducted to establish the scalability of interventions, particularly during and after the COVID19 pandemic when face‐to‐face interventions have been restricted.

## CONFLICT OF INTEREST

The authors have no conflicts of interest to declare.

## Supporting information

Supporting Information S1

## Data Availability

The data that support the findings of this study are available from the corresponding author upon reasonable request.
