# Supplementary material for: Impact of connected health interventions on psychological wellbeing and quality of life in patients with cancer: A systematic review and meta‐analysis
Source: Psychooncology. 2022 Sep 22;31(10):1621–36. doi: 10.1002/pon.6019 (PMC9825891; doi:10.1002/pon.6019)
Supplement: Supplementary file 1 — Supporting Information S1 [file PON-31-1621-s001.docx]

**Supplementary Material**

**Full electronic search strategy**

**Psychinfo:** TX ((Cancer OR Neoplasms) AND TX (Psychology* OR mental health OR Distress OR Depression OR Anxiety OR Sadness OR Posttraumatic stress disorder OR Life satisfaction OR Health related quality of life) AND TX ( Electronic health services OR Smartphone OR Electronic Health Records OR Mobile Applications OR wearable device OR Web OR ehealth or mhealth or app or telehealth ))

**Pubmed**: (("neoplasms"[MeSH Terms] OR "neoplasms"[Title/Abstract] OR "cancer"[Title/Abstract]) OR ("neoplasms"[MeSH Terms] OR "neoplasms"[Title/Abstract])) AND (("psychological distress"[MeSH Terms] OR "psychological distress"[Title/Abstract]) OR ("depression"[MeSH Terms] OR "depression"[Title/Abstract] OR "anxiety"[Title/Abstract]) OR ("anxiety"[MeSH Terms] OR "fear"[Title/Abstract]) OR ("fear"[MeSH Terms] OR "sadness"[Title/Abstract]) OR ("sadness"[MeSH Terms] OR "personal satisfaction"[Title/Abstract]) OR ("personal satisfaction"[MeSH Terms] OR" quality of life"[Title/Abstract]) OR ("quality of life"[MeSH Terms])) AND ("Electronic health services"[Title/Abstract] OR ("telemedicine"[MeSH Terms] OR "telemedicine"[Title/Abstract]) OR ("smartphone"[MeSH Terms] OR "smartphone"[Title/Abstract]) OR "Electronic Health Records"[Title/Abstract] OR ("mobile applications"[MeSH Terms] OR ("mobile"[Title/Abstract] AND "applications"[Title/Abstract]) OR "mobile applications"[Title/Abstract]) OR Web[Title/Abstract] OR ("telemedicine"[MeSH Terms] OR "telemedicine"[Title/Abstract] OR "ehealth"[Title/Abstract]) OR "mhealth"[Title/Abstract]))

**EMBASE**: (cancer:ab,ti OR neoplasm:ab,ti) AND (‘psychological well?being:ab,ti or mental health:ab,ti or ‘distress syndrome':ab,ti or depression:ab,ti or anxiety:ab,ti or ‘fear of recurrence':ab,ti or sadness:ab,ti or ‘posttraumatic stress disorder':ab,ti or ‘life satisfaction':ab,ti or ‘quality of life':ab,ti) AND (telehealth:ab,ti OR mhealth:ab,ti OR smartphone:ab,ti OR 'electronic health record':ab,ti OR 'mobile application':ab,ti OR 'wearable device':ab,ti OR web:ab,ti OR app:ab,ti)

**WEB OF SCIENCE**: #3 AND #2 AND #1. TS= (Cancer or Neoplasm) AND TS= (Psychological well?being OR Mental Health OR psychological distress OR depression OR anxiety OR fear OR sadness OR PTSD OR satisfaction OR quality of life) AND TS= Connected health OR ehealth OR mhealth OR telehealth OR smartphone OR telemedicine OR electronic health record OR app OR wearable OR web) AND TS=(Adults)

**Table 1: Eligibility criteria**

|  | **Inclusionary Criteria** | **Exclusionary Criteria** |
| --- | --- | --- |
| **Population** | Adult diagnosed with cancer (No restriction on type, severity, time since diagnosis, or cancer prognosis, including cancer survivors  An adult is defined as an individual aged 18 years or above. | Cancer patients below 18 years.  Caregivers and families of adult patients with cancer |
| I**ntervention** | Connected health interventions with a measure of psychological wellbeing or quality of life.  Includes smartphones, web‐based interventions, online group‐based interventions, telehealth, and wearables.  No restrictions on the timing of the intervention as long as the intervention is on patients with a confirmed cancer diagnosis. | Interventions that are not connected to the internet.  CH interventions without a measure of psychological outcomes or quality of life.  CH interventions without a comparator |
| **Measures** | Primary data from the patient using validated measures | Secondary data  Measures that have not been validated |
| **Outcomes** | Psychological outcomes or quality of life, with either being the primary outcome.  Psychological wellbeing: Presence or absence of self-reported distress {e.g., anxiety, depression, fear of recurrence, sadness, panic} as well as the presence or absence of self-reported positive constructs such as happiness and satisfaction  Quality of life: Subjective appraisal or evaluation of one’s life, with emphasis on perceived health status and activity limitation. | Outcomes unrelated to psychological wellbeing or quality of life. |
| **Study Design** | All study designs as long as there is some evaluation of CH intervention and the effect of the intervention on psychological outcomes or quality of life was reported. | Literature reviews, systematic reviews, meta-analysis, background articles, commentaries, Descriptive designs |
| **Reporting** | Reports must be in the English language and have appeared in peer-reviewed journals published within the past 10 years. | Reports in non-English languages.  Grey literature  Studies published prior to 2010. |

**Table 2: Study characteristics.**

| **Author** | **Country** | **Cancer type** | **Age (mean)** | **Sample Size** | **Phase** | **Measures** | **Primary outcomes** | **Secondary outcomes** | **Platform** | **Healthcare contact/ Facilitator** |
| --- | --- | --- | --- | --- | --- | --- | --- | --- | --- | --- |
| Owen et al. (2017) | USA | Mixed | 52.9 | 347(176,171) | Survivorship | Distress Thermometer (DT) Outcomes Questionnaire, Profile of mood states, Epidemiologic studies Depression scale, Impact of Events Scale (IES-R) | Distress, psychological functioning, depression, and trauma-related anxiety | Fatigue and Vigor | Website | Psychologists |
| Yun et al. (2020) | South Korea | Stomach, Colon, Lung, and Breast | 54.4 | 394(135,125,134) | Survivorship | Post Traumatic Growth Inventory (PTGI), Hospital Anxiety and Depression Survey (HADS), Brief Fatigue Inventory (BFI) & McGill QoL | Physical Activity, Weight, and positive growth | Anxiety and Depression, Fatigue, social support, and Quality of Life | Website | Nurses |
| Beatty et al. (2015) | Australia | Mixed | 51.6 | 60 (30,30) | Treatment phase | Post-Traumatic Stress Scale- Self Report (PSS-SR), EORTC QOL-C30, and Mini-Mental Adjustment to Cancer Scale(mini-MAC) | Cancer Related Distress | Coping | Website | None |
| Abrahams et al. (2017) | Netherlands | Breast | 52.5 | 132 (66,66) | Survivorship | CIS Fatigue Severity, Sickness Impact Profile, Brief Symptom Inventory, EORTC QOL-C30 | Fatigue severity | Functional Impairment, Psychological Distress, Quality of Life | online CBT | Therapists |
| Lally et al. (2019) | USA | Breast | 55.1 | 100 (57,53) | Treatment phase | DT, Centre for Epidemiologic Studies Depression Scale (CES-D), IES | Cancer Related Distress | NR | Website | None |
| Greer et al. (2019) | USA | Mixed | 56.5 | 145 (72,73) | Treatment phase | Hamilton Anxiety Rating Scale (HAM-A), Clinical Global Impression Scale, HADS, PHQ-9, and Functional Assessment of Cancer Therapy-General | Anxiety | Depression and Quality of Life | mobile | None |
| Stevenson et al. (2019) | Australia | Hematological cancers | 50 | 60 (30,30) | Treatment phase | Health System and Information Needs Domain of the Supportive Care Needs Survey Short Form (SCNS-SF34), HADS | Unmet information needs | Depression and Anxiety | Website | Nurse |
| Sherman et al. (2018) | Australia | Breast | 57.4 | 304 (149,155) | Survivorship | Body Image Scale. Body Appreciation Scale. Self-Compassion Scale–Short Form. Depression, Anxiety, and Stress Scales & Appearance Schemas Inventory-Revised | Body image–related distress (BID) and Body appreciation | Psychological distress (depression and anxiety) and self-compassion | website | None |
| Spahrkäs et al. (2020) | Australia, Canada, the United Kingdom, and the US | Mixed | 55.5 | 799 (519, 280) | Mixed | Fatigue Symptom Inventory [FSI]. EORTC-QLQ-30 | Fatigue severity and Interference | Quality of Life | mobile | None |
| Sui et al. (2019) | China | Lung | 61.4 | 50(100,100) | Treatment phase | HADS. EORTC-QLQ-30 | Depression, anxiety, and QoL | Loss to follow up and survival data analysis | smartphone | Nurses |
| Beatty et al. (2018) | Australia | Mixed | 54.9 | 191 (86,78) | Treatment phase | PSS-SR, EORTC QOL-C30, and Mini-Mental Adjustment to Cancer Scale(mini-MAC), Australian Bureau of statistics Health Service Utilisation Questionnaire | Cancer-specific distress | General distress, (QoL), Coping and Health service utilization | website | None |
| Ruland et (2013) | Norway | Breast and Prostate | 56.7 | 325 (162,163) | Treatment phase | Memorial Symptom Assessment Scale Short Form (MSAS-SF), Global Distress Index (GDI) CES-D 15D HRQoL Instrument. Medical Outcomes Study Social Support Survey | Symptom distress | Depression, self-efficacy, HRQoL, and social support | website | Cancer nurses |
| Zhou et al. (2019) | China | Breast | 49.9 | 111(56,55) | Treatment phase | Functional Assessment of Cancer Therapy-Breast version 4.0 (FACT-Bv4.0) and the Numerical Rating Scale | Health-related quality of life | Pain, fatigue, and sleep | smartphone | Nurses and doctors |
| Korkmaz et al. 2019 | Turkey | Breast | 47.8 | 72 (24,24,24) | Treatment phase | Risk Factors for Breast Cancer and Data Collection Form for the Disease, SF 36 QoL Scale, State-Trait Anxiety Inventory, and Website Usability Scale | Anxiety and Quality of Life | NR | Website | None |
| Bruggerman- Everts et al. (2017) | Netherlands | Breast | 56.3 | 167(62,55, 50) | Survivorship | Checklist Individual Strength - Fatigue Severity, HADS. Positive and Negative Affect Schedule | Self-perceived fatigue severity | Mental Health | Website | Psychologist |
| Willems et al. (2017) | Netherlands | Mixed | 55.86 | 462 (231,231) | Survivorship | EORTC QoL, HADS, Checklist Individual Strength (CIS) | Emotional and Social functioning, Depression and Fatigue | NR | website | None |
| Rosen et al. (2018) | USA | Breast | 52.31 | 112 (57, 55) | Treatment phase | Functional Assessment of Cancer Therapy—Breast version 4 (FACT‐B). Mindful Attention Awareness Scale (MAAS) | Quality of Life | Dispositional Mindfulness | smartphone | None |
| Kuhar et al. (2020) | Slovenia | Breast | 51.7 | 91(46,45) | Treatment phase | EORTC C-30, BR- 23 Breast Cancer Questionnaires | Global quality of life | Use of health resources (doctor visits and hospitalizations) | smartphone | Oncologist |
| Vallance et al. (2020) | Canda and Australia | Breast | NR | 83 (43,40) | Survivorship | Actigraph and activPAL accelerometers. Functional Assessment of Cancer Therapy-Breast (FACT-B) and the Functional Assessment of Chronic Illness Therapy Fatigue (FACIT-Fatigue) | Physical activity | HRQoL and fatigue | Wearable | Health promotion experts/Kinesiology |
| Anja van der Hout et al. (2020) | Netherlands | Multiple (head and neck cancer, colorectal cancer, breast cancer, Hodgkin lymphoma, or non-Hodgkin lymphoma) | 65 | 625 (320,305) | Survivorship | Patient Activation Measure. EORTC QLQ-C30. The mental adjustment to cancer scale. The Supportive Care Needs Survey Short Form 34. The General Self-Efficacy. The Pearlin & Schooler Mastery Scale. The Perceived Efficacy Patient-Physician Interactions scale. The Functional, Communicative, and Critical Health Literacy scale. The Multidimensional Health Locus of Control. The eHealth Impact Questionnaire | Patient activation (knowledge, skills, and confidence for self-management) | HRQoL, mental adjustment to cancer, supportive care needs, self-efficacy, personal control, and perceived efficacy in patient-physician interaction | website | specialists |
| Fjell et al. (2019) | Sweden | Breast | 46 | 149 (74,75) | Treatment phase | Memorial Symptom Assessment Scale (MSAS). EORTC QLQ-C30 | Symptom burden | HRQoL | smartphone | Nurses |
| Sundberg et al. (2017) | Sweden | Prostate | 69 | 110(66, 64) | Treatment phase | EORTC QLQ-C30.Sense of Coherence questionnaire | Symptom burden | HRQoL | smartphone | Nurses |
| Compen et al. (2018) | Netherlands | Mixed | 51.65 | 245(90,77, 78) | Treatment phase | HADS. Structured Clinical Interview for DSM-IV-TR Axis I Disorders. Fear of Cancer Recurrence Inventory. Rumination and Reflection Questionnaire. mental and physical scales of the Short-Form 12. Five Facet Mindfulness Questionnaire-Short Form. Mental Health Continuum-Short Form. Neuroticism Extraversion Openness-Five Factor Inventory | Psychological distress | Psychiatric diagnosis, fear of cancer recurrence, rumination, health-related quality of life, mindfulness skills, and positive mental health. | website | Therapists |
| Syrjala et al. (2018) | USA | Haematological cancers | 52 | 755 (344, 411)51 | Survivorship | Cancer and Treatment Distress (CTXD), Symptom Checklist-90-R depression scale (SCL-90-R), Short Form 36 Health Survey (SF-36), and Fatigue Symptom Inventory (FSI) | Cancer Related Distress | Fatigue, depression, and general health | website | Ph.D. level psychologists |
| Ridner et al. (2019) | USA | Breast | 56.8 | 160 (80,80) | Survivorship | Lymphedema Symptom Intensity and Distress Scale–Arm (LSIDS-A). Profile of Mood States-Short Form (POMS-SF). Quick-Disabilities of Arm, Shoulder, and Hand (QuickDASH). Perceived Medical Condition Self-Management Scale. Medical Outcomes Study Social Support Survey (MOS Social Support Survey). Resource Utilization and Economic Burden Questionnaire (RUEBQ) | Symptom burden, psychological well-being, function, and costs, and arm volume | NR | website | None |
| Helmondt et al. (2019) | Netherlands | Breast | 55.8 | 262 (130,132) | Survivorship | Fear of Cancer Recurrence Inventory (FCRI‐SF‐NL). Psychosocial Distress Questionnaire‐Breast Cancer (PDQ‐BC) | Fear of cancer recurrence | Coping strategies, functioning impairments, and psychological distress | web | None |
| Chambers et al. (2018) | Australia | Mixed | NR | 163 (79,84) | Treatment | Brief Symptom Inventory. The Impact of Event Scale. Supportive Care Needs Survey Short Form. Posttraumatic Growth Inventory. Assessment of Quality-of-Life 8D. The Internet Evaluation and Utility Questionnaire and internet Intervention Adherence Questionnaire (Process Measures) | Psychological and cancer-specific distress and unmet psychological supportive care needs | Positive adjustment and quality of life. | Web-based CBT | Psychologists |
| Hou et al. (2020) | Taiwan | Breast | 52 - 64 (Range) | 112 (53, 59) | Active Treatment | EORTCQLQ-C30). EORTC Breast Cancer-Specific QOL (QLQ-BR23) | Quality of Life | NR | mobile app | Psychologists |
| Hauffman et al. (2020) | Sweden | Breast, colorectal, prostate cancer | 59 | 15 | Mixed | Interview Guide | Anxiety and Depression | NA | Web-based program | Psychologists |
| Li and Di (2018) | China | Nasopharyngeal Carcinoma | 44.3 | 132 (65,67) | Treatment | EORTC QOL | complications and quality of life | NR | smartphone | Oncologist |
| Admiraal et al. (2017) | Netherlands | Breast | 53.2 | 139 (70, 69) | Survivorship | EORTC QOL. Breast cancer-specific QOL(QLQ-BR23) Constructs empowering outcomes (CEO). DT, and 47-item Problem List (PL) | Optimism and control over the future, feeling informed, and acceptance of the illness | Distress and quality of Life | Web-based program | Psychologists |
| Willems et al. (2017) | Netherlands | Mixed | 56.3 | 462 (231,231) | Survivorship | EORTCQL-C30. HADS. Checklist Individual Strength | QoL, anxiety, depression, and fatigue | NR | website | None |
| Mayer et al. (2018) | USA | Colon | 58.6 | 284 (140,144) | Survivorship | Godin Leisure-Time Physical Activity Questionnaire (GLTPAQ). Functional Assessment of Cancer Therapy-Colon (FACT-C, version 4). NCCN Distress Tool. Treatment Self-Regulation Questionnaire [TSRQ]. McTavish Bonding Scale | Physical Activity | Distress and Quality of Life | SMARTPHONE | Coaches |
| Greer et al. (2020) | USA | Mixed | 53.3 | 181 (91,90) | Active Treatment | Electronic Pill Caps, MD Anderson Symptom Inventory (MDASI). Functional Assessment of Cancer Therapy–General (FACT-G). Morisky Medication Adherence Scale (MMAS-4). Functional Assessment of Chronic Illness Therapy– Treatment Satisfaction–Patient Satisfaction (FACIT-TSPS) | Medication adherence, Symptom burden, and QoL | Anxiety and depression, social support, quality of care, and healthcare utilization | mobile app | None |
| Freeman et al. (2015) | USA | Breast | 55.4 | 118(23,48, 47) | Survivorship | Medical Outcomes Study 36-item short-form survey (SF-36). Functional Assessment of Cancer Therapy-Breast (FACT-B). FACIT-Fatigue Scale (FACIT-F, version 4). FACT-Cog (version 2). Functional Assessment of Chronic Illness Therapy Spiritual Well-Being Expanded Scale (FACIT-Sp-Ex; version 4). Brief Symptom Inventory (BSI-18) Global Severity Index (BSIGSI). Pittsburgh Sleep Quality Index (PSQI) | Health-related and breast cancer-specific QOL | Fatigue, cognitive function, spirituality, distress, and sleep | tele delivery/live streaming | Therapist |
| Yun et al. (2012) | South Korea | Mixed | NR | 273 (136,137) | Survivorship | Brief Fatigue Inventory (BFI). Fatigue Severity Scale (FSS). HADS. EORTC QLQ-C30 | Cancer-related Fatigue | Anxiety, depression, and Quality of Life | website | Health professional -Not specified |
| Basch et al. (2016) | USA | Mixed | 61.5 | 766 (539, 227) | Treatment | EuroQol EQ-5D Index | HRQoL | Emergency Room visits, Hospitalization, and survival. | Web-based interface | Nurses and oncologists |
